# Well-Being and Coping Capacities of Adolescent Students with Hearing Loss in Mainstream Schools

**Published:** 2020

**Authors:** Narges ADIBSERESHKI, Nikta HATAMIZADEH, Firoozeh SAJEDI, Anoshirvan KAZEMNEJAD

**Affiliations:** 1 Department of Psychology and Education of Exceptional Children, University of Social Welfare and Rehabilitation Sciences, Tehran, Iran; 2 Pediatric Neurorehabilitation Research Center, University of Social Welfare and Rehabilitation Sciences, Tehran, Iran.; 3 Department of Biostatistics, Faculty of Medical Sciences, Tarbiat Modares University, Tehran, Iran.

**Keywords:** Adolescents with hearing loss, Well-being, Coping strategies

## Abstract

**Objectives:**

Coping strategies used by adolescents has an important role in preventing or decreasing their stresses and also increasing their well-beings. This study aimed at evaluating the coping capacity and well-being of adolescent students with hearing loss in mainstream schools and also the correlations between their coping strategies and positive characteristics of well-being (engagement, perseverance, optimism, connectedness and happiness (EPOCH).

**Materials & Methods:**

In this correlational study, 122 adolescent students with hearing loss were randomly selected from mainstream schools. Data collection was done by EPOCH Measure of Adolescent Well-Being and the Ways of Coping Questionnaire (WAYS). The Spearman correlation coefficient was used for determining the correlations between variables.

**Results:**

The mean scores of using different coping strategies varied from 1.36 in problem solving to 1.44 in seeking support. Among the positive characteristics of well-being, happiness had the lowest (11.04) and connectedness showed the highest score (12.33). The findings also showed a significant correlation between all coping strategies and EPOCH, however there was a strong positive correlation between total coping strategy score and perseverance (0.648) and happiness (0.629).

**Conclusion:**

Based on the results, the score of happiness in students with hearing loss was the lowest among positive characteristics of well-being and also happiness showed a strong association with total scores in coping strategies. Accordingly, interventional studies are needed to examine whether training students with hearing loss to use coping strategies is effective in increasing their happiness and overall well-being.

## Introduction

Adolescence is a period of significant changes in biological, psychological, socio-emotional and cognition development that can be associated with behavioral, social and health problems (1). Through this period, people are more exposed to increased rates of stressful situations than their childhood. There are several strategies to deal with stress, which are called coping styles (2). They have been categorized as problem-focused versus emotion-focused coping styles (3) as well as approach versus avoidance coping styles (4). The most commonly used coping strategies by adolescents are seeking social support, problem solving, avoidance coping, palliative emotion regulation and anger-related emotion regulation (5). Stress is one of the factors that negatively affect individual’s well-being. There are some evidences that increase in stressors is a potential threat to the well-being and healthy development of adolescents (6). Well-being refers to the healthy functioning (physiological, psychological and behavioral), positive social relationships and a safe social life (7). Seligman (8) states that positive emotions, engagement, positive relationships, meaning and accomplishment are the most significant factors associated with the overall sense of well-being in individuals. Based on Seligman’s (8) theory, five factors have reported effective on well-being during adolescence, including engagement (focused on what an individuals is doing and interested through his life), perseverance (pursuing goals to accomplish), optimism (being hopeful and looking forward to the future), connectedness (positive relationships with others) and happiness (positive affect), which are called EPOCH (9).

Coping strategies mediates the relationship between predictor measures of functional limitations and perceived visibility of condition and outcome measures of disability-specific psychosocial adjustment and life satisfactions of people with disability (10). Livneh and Antonak examined hearing loss as an acquired and also as a congenital disability (11). The acquired and unexpected disability, such as late onset hearing loss causes the individuals to experience more stresses, losses, grief and stigmas. However, they state that hearing loss as a chronic illness and disability (CID) limits the individuals to engage in daily life activities. Several CID hearing loss cases can be degenerative, which means that the individual’s performance can be declined overtime. Stress, adaptation, quality of life and especially psychological well-being are the great concerns for those with this condition. Therefore, continuous adaptation and interventions to facilitate coping seems very important.

Adolescents with hearing loss are often faced with the additional challenge of adapting to hearing world, where communication and also access to information is difficult (12). Those enrolled in mainstream schools (regular schools including students with disability) have the additional challenge with their hearing peers, many of whom may not share a language with them (12). Brunnberg, Boström and Berglund (13) found more mental symptoms and school problems in adolescents with hearing impairment compared to their normal hearing classmates. 

Exposure to different stressors is typically associated with low levels of well-being (14). Children with hearing loss may experience higher level of stress (15) and lots of problems regarding school and greater psychosocial difficulties, such as internalizing symptoms (16). Problems while using a hearing aid, amplitude and duration cues, communication and education problems impose additional stresses to their lives (17, 18). Children with hearing loss showed lower scores for problem solving, higher scores for coping strategies, including anger-related emotion regulation, seeking social support (girls) and using media (boys) (17).

Psychologists have always been interested in the risk and/or supportive factors that impact individual’s well-being. Studies (19) on coping strategies in adolescents are focused on how this group cope with negative emotions (worry, sadness, anger, helplessness and pessimism). Investigating how adolescents cope with their emotions is of great importance, however coping strategies may be more important than the emotions themselves to affect well-being (20). In children with hearing loss, everyday stressors (such as being communicated with others) are experienced as more stressful than normal hearing pupils. They have problem in using some of coping strategies (17), which can result in well-being impairments. 

Based on relevant studies, using coping strategies has a significant relationship with well-being (21). On the other hand, the state of well-being and coping capacity among adolescent with hearing loss in mainstream schools have not well defined. The objectives of the present study was to determine the coping capacity and well-being of adolescent students with hearing loss in mainstream schools and also the correlations between use of coping strategies and positive characteristics of well-being (EPOCH). The results can provide evidences on the coping strategies and well-being in adolescents with hearing loss and whether using different coping strategies by adolescent with hearing loss in facing stress can predict their positive characteristics of well-being.

## Materials & Methods

Participants

The study population consisted of 122 students with hearing loss. They were recruited from mainstreaming schools (studying in the 6^th^ to 9^th^ grades). The samples consisted of 48 girls and 74 boys, of whom 22 samples (18%) were studying in the 6^th^ grade, 24 samples (19.67%) in the 7^th^, 51 samples (41.8%) in the 8^th^ and 25 samples (20.5%) in the 9^th^ grades. With no hearing aid, the severity of hearing loss in better ear in 20 subjects (16.4%) was mild, in 85 subjects (69.7%) was moderate and in 17 participants (13.9%) was severe. Using hearing aid, the corrected hearing thresholds in better ear of 83 participants (68%) was within the mild, in 24 subjects (19.7%) was in the moderate and in 15 subjects (12.3%) found in the severe range of hearing loss. Of all participants, 78 cases (51.6%) used hearing aid in one ear, 44 cases (36.1%) in both ears and 8 subjects (6.6%) had cochlear implants. Two students (1.6%) had a parent with hearing loss. About 94 students (77%) had been started to use hearing aids at the ages of 0-4 years and 28 students (23%) from the ages of 5-10 years. In addition, 2 students (1.6%) had cochlear implant when they were 2 years old, 4 students (3.2%) at the age of 3 years and 2 cases (1.6%) at the age of 4 years. The cause of hearing loss for 106 participants (86.89%) was congenital and 16 subjects (13.11%) reported the acquired hearing loss. 

*Instruments*

*1) Demographic questionnaire*. A brief demographic questionnaire was used to collect the information, such as gender, grade, using hearing aid and cochlear implant.

*2) The EPOCH Measure of Adolescent Well-Being*. This is a 20-item scale based on the well-being theory and assesses 5 positive psychological characteristics of well-being, including engagement, perseverance, optimism, connectedness and happiness (9). A 5-point Likert scale ranged from never (1) to always (5) was used to answer the items. The total score for each positive characteristic is consisted of sum of scores of related items. Higher scores are representative of higher levels of well-being. In present study, the Cronbach's alpha coefficient of the EPOCH for students with hearing loss was obtained 0.749.

*3) The Ways of Coping Questionnaire: *This is a 66-item questionnaire assessing thoughts and behaviors used by people to deal with stress (3). The items are grouped in 8 ways/strategies of coping, including confrontive coping (6 items), distancing (6 items), self-controlling (7 items), seeking social support (6 items), accepting responsibilities (4 items), escape-avoidance (8 items), painful problem solving (6 item) and positive reappraisal (7 items). The participants are asked to report the extent, to which they use each strategy and the questions are scored on a 4-item Likert scale with scores ranging from 0 to 3 as follows: 0 (not used), 1 (used somewhat), 2 (used quite a bit) and 3 (used a great deal). In present study, the Cronbach's alpha coefficient for students with hearing loss was found 0.905.

SPSS version 21 was used for statistical analysis. The means and standard deviations and Spearman correlation coefficients were calculated for data analysis. P<0.05 was considered as statistically significant.

## Results

The study population consisted of 122 adolescent students with hearing loss. The well-being of the adolescent students with hearing loss who were studying in the regular schools was measured by EPOCH Measure. The results are shown in Table 1. 

**Table 1:** The mean and min-max scores for well-being of adolescents with hearing loss in the mainstreaming schools

		Mean±SD	Min-Max
Well-being (positive characteristics)	Engagement	11.70± 2.84	6-18
	Perseverance	11.60± 2.63	5-20
	Optimism	11.65± 2.78	7-18
	Connectedness	12.33± 3.16	6-19
	Happiness	11.04± 0.84	5-20

Based on the EPOCH, the scores for each positive psychological characteristic are ranged from 5 to 20.

As shown in Table 1, the mean scores for the five positive psychological characteristics ranged from 11.04 for happiness to 12.33 for connectedness in a 5-20 grading scale.

The mean scores of coping strategies in adolescents with hearing loss in the mainstreaming schools

are shown in Table 2.

**Table 2:** The mean scores of coping strategies in adolescents with hearing loss in the mainstreaming schools

Coping strategies	Mean±SD	Min-Max
Confronting	1.40±0.68	0-3
Distancing	1.40±0.67	0-3
Self-control	1.39±0.70	0-3
Seeking support	1.44±0.69	0-3
Accept responsibility	1.34±0.82	0-3
Escape-avoidance	1.27±0.63	0.13-2.88
Problem solving	1.36±0.68	0-3
Positive reappraisal	1.30±0.67	0-3
Cop total	1.38±0.53	0.52-2.83

According to the table 2, the mean scores varied from 1.36 for problem solving strategy to 1.44 for seeking support.

The correlations between positive characteristics of the adolescents with hearing loss and using various coping strategies are demonstrated in table 3.

**Table3:** The correlations between five positive psychological characteristics of well-being (EPOCH) and using various coping strategies in adolescents with hearing loss

Coping Strategies	Confronting	distancing	Self-control	Seeking support	Accept responsibility	Escape Avoidance	Problem Solving	Positive Reappraisal	Cope total score
Positive characteristics									
Engagement	0.368*	0.261 (p=0.004) ^†^	0.471	0.314	0.374	0.398	0.524	0.335	0.503
Perseverance	0.547	0.411	0.547	0.427	0.462	0.602	0.591	0.522	0.648
Optimism	0.431	0.424	0.433	0.556	0.393	0.386	0.474	0.493	0.529
Connectedness	0.425	0.263 (p=0.003)	0416	0.429	0.384	0.411	0.437	0.364	0.453
Happiness	0.553	0.441	0.511	0.566	0.466	0.493	0.593	0.553	0.629

*Spearman correlation coefficients are shown in whole table,

^† ^P value < 0.001.

As it can be seen in Table 3, there were significant positive correlations between all coping strategies and all five positive characteristics of well-being (EPOCH). The Spearman correlation coefficients were more than 2.6 in all circumstances, and the greatest correlations were seen between two positive characteristics, including perseverance (0.648) as well as happiness (0.629) and the total coping strategy score.

## Discussion

This study was done to assess the coping capacity and well-being of adolescents with hearing loss and also to determine whether different coping strategies are correlated with EPOCH.

Regarding the first objective, the results of this study revealed that in students with hearing loss, the mean scores for using different coping strategies ranged from 1.36 in using problem solving to 1.44 in seeking support strategies. Eschenbech et al. study (17) indicated that children with hearing loss scored higher than hearing children in seeking social support. They stated that children with hearing loss experience stresses of everyday life in the family, school and while being with peers more than normal hearing children, which can lead to problems with using some of the coping strategies. When adolescents with hearing loss are faced with stress, they adopt different coping strategies according to their interaction and communication (especially pragmatics) skills (22).

This study investigated the positive characteristics of well-being (EPOCH) in adolescent students with hearing loss. We found no study on EPOCH in this goup; however some studies have addressed other factors associated with poor mental health and psychological well-being of individuals with hearing loss. For example, in a study (23) YSR (Achenbach adaptive function and behavior measures) was used on 89 students with hearing loss and it was revealed that 40% of the respondents reported internalizing problems and 37% announced externalizing problems, somatic complaints, rule breaking behavior, attention problems, aggressive behavior, social problems and also withdrawn, anxiety/depression and thought problems, respectively. Our results indicated that in students with hearing loss, the highest score for positive characteristics of well-being is recorded for connectedness and the lowest is recorded for the happiness. Some studies reported the highest score for connectedness, whereas the lowest score is noted for engagement (24) in students with normal hearing. There is a strong link between social, behavioral and communicational problems and mental health. Development of communication skill is essential for family, school and social relationships (connection with others) and this may be problematic for children with hearing loss resulting in communication (25), social, emotional and mental well-being implications (23). For example, school is where adolescents spend lots of their time and they experience positive relationships in schools. A sense of belonging at school is associated with the classroom engagement and academic motivation and is an effective factor for well-being (26). Moreover, friendships and doing activities with friends are sources of happiness in a child’s life, in which the child with hearing loss is faced with several problems. It seems that communication difficulties lead to connectedness and friendship problems and impact the happiness and well-being of individual with hearing loss.

For the second objective, the results of this study showed significant correlations between coping strategies and EPOCH. Although the relationship between overall well-being and using coping strategies has been studied, the researchers have used different measurement tools. Ryff (27) and Keyes (28) measured subjective well-being and their results showed that the higher level of well-being was associated with higher use of coping strategies in youth (29). The findings of this study Students with hearing loss the strong associations between optimism and coping strategies (seeking social support and positive reappraisal more than others), connectedness and coping strategies (seeking social support and problem solving more than others), happiness and coping strategies (problem solving, seeking social support more than others). Higher levels of social connectedness and social support are associated with better physical and mental health outcomes (30). Social connectedness and social support act as the protective factors against stress, by which individuals under stressful situation can use seeking social support as a coping strategy to face the stress (31). Normal hearing individuals with high social support might be more optimistic and can better cope with stressful situations, which can lead to more positive health outcomes (32). Studies on happiness indicated that coping strategies influence happiness. Being in a group is associated with happiness for adolescents (33, 34), where social support and coping have a proper association with well-being in them (35). Our finding also revealed that two positive characteristics of wellbeing, including engagement and perseverance were more strongly associated with problem solving coping strategy than other coping strategies in adolescent with hearing loss. Problem solving is engaging in a task, for which there is no known solution and the students must persist on the task to be able to solve the problem. Adler (36) indicated that in three studies on well-being in secondary school students, perseverance, engagement and quality of relationships were strongest mechanisms for increasing well-being and academic performance. Perseverance leads to behaviors, such as attending class, doing homework, engaging in classroom activities and studying, which are closely linked to academic success (37).

This study had some limitations. We did not consider some differences, such as differences in oral communication skills of students. The population of people with hearing loss is diverse and they have different needs. Our findings cannot be generalized to all subjects with hearing loss. 

**In Conclusion,**The results showed that the present status of positive characteristics of well-being (engagement, perseverance, optimism, connectedness, happiness) and coping strategies (confronting, distancing, self-controlling, seeking social support, accepting responsibilities, escape-avoidance, planful problem solving and positive reappraisal) among adolescent students with hearing loss. The findings regarding coping strategies and EPOCH can add to the information in this field and also it can be effective for educators and professionals to design interventional programs for adolescents with hearing loss.
